# Identification of countercurrent tubule-vessel arrangements in the early development of mouse kidney based on immunohistochemistry and computer-assisted 3D visualization

**DOI:** 10.1371/journal.pone.0307223

**Published:** 2024-08-13

**Authors:** Yun-Sheng Ma, Si-Qi Deng, Ping Zhang, Jesper Skovhus Thomsen, Arne Andreasen, Shi-Jie Chang, Jie Zhang, Ling Gu, Xiao-Yue Zhai

**Affiliations:** 1 Department of Histology and Embryology, Basic Medical College, China Medical University, Shenyang, Liaoning, China; 2 Department of Morphology, Medical College of Jinzhou Medical University, Jinzhou, Liaoning, China; 3 Department of Pathology, the Fourth Affiliated Hospital of China Medical University, Shenyang, Liaoning, China; 4 Department of Biomedicine–Anatomy, Aarhus University, Aarhus, Denmark; 5 Department of Biomedical Engineering, School of Intelligent Medicine, China Medical University, Shenyang, Liaoning, China; 6 Institute of Nephropathology, China Medical University, Shenyang, Liaoning, China; University of Bari Aldo Moro: Universita degli Studi di Bari Aldo Moro, ITALY

## Abstract

Nephron loop-vessel countercurrent arrangement in the medulla provides the structural basis for the formation of concentrated urine. To date, the morphogenesis of it and relevant water and solutes transportation has not been fully elucidated. In this study, with immunohistochemistry for aquaporins (AQP) and Na-K-2Cl co-transporter (NKCC2), as well as 3D visualization, we noticed in embryonic day 14.5 kidneys that the countercurrent arrangement of two pairs of loop-vessel was established as soon as the loop and vessel both extended into the medulla. One pair happened between descending limb and ascending vasa recta, the other occurred between thick ascending limb and descending vasa recta. Meanwhile, the immunohistochemical results showed that the limb and vessel expressing AQP-1 such as descending thick and thin limb and descending vasa recta was always accompanied with AQP-1 negative ascending vasa recta or capillaries and thick ascending limb, respectively. Moreover, the thick ascending limb expressing NKCC2 closely contacted with descending vasa recta without expressing NKCC2. As kidney developed, an increasing number of loop-vessels in countercurrent arrangement extended into the interstitium of the medulla. In addition, we observed that the AQP-2 positive ureteric bud and their branches were separated from those pairs of tubule-vessels by a relatively large and thin-walled veins or capillaries. Thus, the present study reveals that the loop-vessel countercurrent arrangement is formed at the early stage of nephrogenesis, which facilitates the efficient transportation of water and electrolytes to maintain the medullary osmolality and to form a concentrated urine.

## Introduction

In kidneys, water and electrolytes are efficiently exchanged between the ultrafiltrate in tubules and plasma in vessels, attributing to the intimate arrangement of the tubules and peritubular capillaries in the cortex, as well as the countercurrent arrangement of the straight loops of Henle with the vasa recta in the medulla.

In the 1970s, Kriz described that in the outer medulla, the descending thin limbs (DTL) of short looped nephrons (SLN) ran in close contact with the so-called vascular bundle where the descending vasa recta (DVR) and ascending vasa recta (AVR) ran in parallel. In humans and rats, DTL of SLN ran at the periphery of the vascular bundle, while in mice, they were integrated into the outer part of the vascular bundle [1–4]. The DTL and vessels running in a countercurrent form lays the structural basis for the urine-concentrating mechanism first proposed in 1942 by Kuhn and Ryfell, i.e. the pair of countercurrent tubules and vessels works as a multiplier by exchanging water and electrolytes, generating the osmotic gradient in the outer medulla [5].

In recent years, the combination of 3D reconstruction techniques, mathematical models, and immunohistochemical labeling for membrane transporters, has largely improved our understanding of the architecture of medulla and its physiological function [4, 6–9]. Among these transporters, water channels, aquaporins (AQP) -1 and AQP-2, and ion channel Na-K-2Cl co-transporter (NKCC2), play essential roles in forming the increasing gradient osmolality towards the medullary apex and concentrated urine at the tip [9, 10]. However, the questions of how and when the countercurrent tubule-vessel arrangement is established and whether it is related with the establishment of water transportation mechanism have not been completely answered, yet.

Kidney morphogenesis is initiated with the invasion of the ureteric bud (UB) derived from the nephric duct into the metanephros. As the UB grows and branches repeatedly outward to the renal capsule, the nephron progenitor cells are induced by the terminal UB tips in nephrogenic zone beneath the renal capsule to experience sequent transformations and eventually differentiate into the mature nephron [11]. Due to the successive nephrogenesis through the embryonic and first postnatal days [12, 13], the Henle loops sequentially extend into the medulla, parallel to the UB branches, i.e. the collecting duct system in adult kidney, consequently forming the outer medulla and long, narrow inner medulla.

The aim of the present study was to investigate the relationship between the nephron loops and vessels and the involvement of water and ions transporters along them during mouse kidney development by immunohistochemistry and the digital tubule tracing technique.

## Materials and methods

### Animals

Kunming mice were raised under specific pathogen-free conditions. The day when the cervical mucus plug was observed was designated as embryonic day (E) 0.5, and the day at birth was designated as postnatal day (P) 0. Prenatal kidneys were obtained from E14.5, E15.5, E16.5, E17.5 and E18.5 fetuses, whereas postnatal kidneys were obtained from P1, P3, P5 pups, as well as P28, P40, and P56 adult mice. At each time point, at least three mice were euthanized for the study without specific gender consideration. The animal experiments were performed in accordance with the code of ethics of the World Medical Association and were approved by the Medical Ethics Committee of China Medical University.

### Paraffin section preparation for immunohistochemistry and immunofluorescence studies

As described earlier [14–16], fetal kidneys were removed after injecting 1% pentobarbital sodium (40 mg / kg body wt) into the peritoneal cavity of pregnant mice and then were fixed by 4% paraformaldehyde in 0.01 M phosphate-buffered saline (PBS, pH 7.4) overnight. Kidneys from P1 mice were immersion-fixed, and P5, P28, P40, and P56 mice were perfusion-fixed through the heart with the above-described fixative. Kidneys were cut into blocks that contained cortex and medulla, and the blocks were further fixed overnight at 4°C in the same fixative, dehydrated in ascending grades of ethanol concentration (70%, 80%, 90%, 95%, and 100%) in sequence, cleared in xylene (at 25°C) for a varied period ranging from several seconds for fetal kidneys to minutes for the pup and the adult, embedded in wax (melting point 56–58°C), and naturally cooled at room temperature to paraffin blocks. Five-μm-thick coronary sections were cut on a microtome (Leica RM-2245).

### Epoxy or paraffin serial section preparation for tubular tracing

Paraffin serial sections were obtained from E14.5 and P5 kidneys, while the epoxy serial sections were from E17.5 and P56 kidneys, as described previously [14–16]. The fixation process of E14.5 and P5 kidneys was the same as described above. After anesthesia, the kidneys of E17.5 fetus were removed and immersion fixed by 1% glutaraldehyde (0.06 M sodium cacodylate buffer and 4% hydroxyethyl starch). Kidneys from P56 were removed after perfusion-fixation through the abdominal aorta with the above-described solution. Kidneys or tissue blocks were dehydrated and then embedded in either paraffin wax (E14.5 and P5) or EPON 812 (E17.5 and P56). Finally, five-μm-thick serial paraffin sections were obtained from E14.5 (530 sections) and P5 (413 sections) kidneys with a microtome (Leica RM-2245); 2.5-μm-thick serial epoxy sections were obtained from E17.5 (700 sections) and P56 (2500 sections) kidneys using a microtome (Reichert, Vienna, Austria) and a diamond knife (Diatome, Biel, Switzerland). In order to identify the microstructure of renal tissue, we stained epoxy sections with toluidine blue and paraffin sections with hematoxylin and eosin (HE).

### Immunohistochemical staining for aquaporins

After removing the paraffin with xylene and rehydrating with descending grades of ethanol concentration (100%, 95%, 90%, 80%, and 70%), the tissue sections were immersed with 0.3% H_2_O_2_ to block the endogenous peroxidase activity. The target antigens in the tissue were retrieved by steam heating (120°C, 100 Kpa) for 2.5 min in citrate buffer (10 mM sodium citrate, pH 6.0). After thoroughly cooled down to the room temperature, the sections were preincubated for 30 min in 0.01 M PBS (1% bovine serum albumin, 0.05% saponin, and 0.05 M glycine, pH 7.4). The sections derived from fetal kidneys were then incubated overnight at 4°C with primary antibodies overnight. The concentration of primary antibodies increased with the kidney development. The rabbit anti-AQP-1 antibody (# AB2219, Millipore, Massachusetts, USA) was diluted from 1:50 for fetal kidneys to 1:200 for pup and adult kidneys. The rabbit anti-AQP-2 antibody (# A7310, Sigma-Aldrich, St. Louis, USA) was diluted from 1:50 for fetus and 1:200 for the pup, to 1:400 for the adult. After washing three times in 0.01 M PBS, the sections were incubated with 1:200 HRP-conjugated goat anti-rabbit IgG (# S0001, Affinity Biosciences, Cincinnati, USA) for 1 h at room temperature. The reaction for immunoperoxidase was visualized with a diaminobenzidine kit (# ZLI-9017, ZSGB-BIO, Beijing, China) for 5 minutes or so, dependent on antibody concentrations for different developing kidneys. Finally, the embryonic and postnatal kidney sections were counterstained for 15 s and 25 s, respectively, with hematoxylin, washed for 20 min in running water, dehydrated in upgraded ethanol, cleared in xylene, and mounted with Eukitt® (O. Kindler, Freiburg, Germany). Images were recorded at × 40 using a microscope (Nikon 80iBF-F-P, Tokyo, Japan) equipped with a Nikon DS digital camera.

All negative controls were carried out paralleled by withdrawing the primary antibodies out of the incubation PBS buffer (0.1% bovine serum albumin, 0.05% saponin, and 0.05 M glycine, pH 7.4). All positive controls were performed in adult kidney sections [9].

### Double labeled immunofluorescent staining for SLC12A1 and AQP-1

The double-labeled immunofluorescent staining was performed in two separated antibody incubation steps, since the two primary and secondary antibodies were both derived from same species, i.e. rabbit and goat, respectively. The process of dehydration and antigen retrieval of paraffin sections was consistent with that described above. Before incubating with each primary antibody, the sections were pre-incubated for 30 minutes at room temperature in PBS containing 5% bovine serum albumin. The renal sections of fetus, pups, and adult were incubated overnight at 4°C with rabbit anti-AQP-1 antibodies (diluted to 1:50, 1:200, and 1:400 respectively), followed by Alexa Fluor® 488 AffiniPure Fab Fragment goat anti-rabbit IgG (H+L) (diluted 1:200, # 111-547-003, Jackson ImmunoResearch Laboratories, West Grove, USA). After the renal sections of fetus, pups and adult were thoroughly washed in PBS, they were incubated overnight at 4°C with rabbit anti-SLC12A1 (diluted 1:50, 1:50, 1:100 respectively, # PA5-80002, Thermo Fisher Scientific, Waltham, USA), followed by goat anti-rabbit IgG (TRITC) (diluted 1:200, # ab6718, Abcam, Cambridge, UK). After washing in PBS, they were mounted with antifade mounting medium (# P0126; Beyotime, Haimen, China) and then examined with a confocal microscope (Nikon CI plus, Tokyo, Japan). Detection of each antigen was carried out sequentially when double labeling was performed using two primary antibodies from the same host species as described in detail [17, 18].

All negative and positive controls were carried out as described above.

### Image recording and alignment

Images of the serial epoxy sections from E17.5 and the adult kidneys were obtained by the “PC‑Microscope‑Digital Camera” image acquisition system (Olympus AX 70 Microscope, Olympus DP 50 Digital Camera, Tokyo, Japan). Four partly overlapping digital images from each section were registered and stitched into one 24-bit color image using analySIS (Version 3.2, Soft Imaging System, Münster, Germany). In addition, the serial paraffin sections from E14.5 and P5 were digitized with a high-resolution digital slide scanner (NanoZoomer 2.0 Digital Pathology, Hamamatsu Photonics, Hamamatsu, Japan). With respect to the quality, the improper image stacks were corrected by running the aligning procedure iteratively with a set of initial conditions until the alignment converted into global optimum [15, 16, 19]. The final resolutions of the images from E14.5, E17.5, P5, and the adult kidney were 0.92, 1.0, 1.83, and 1.16 μm/pixel, respectively.

### Digital tracing and three-dimensional representation

The tracing of renal tubules and blood vessels was performed by a custom-made computer program running on the Linux-based PC [20]. In brief, markers were put manually in the lumen of tubules or vessels, each of which corresponded to an x−, y−, and z‑coordinates. A set of coordinates depicting the course were automatically recorded in a data file, which can be inputted into a rotatable 3D plot for visualizing the path in space. Tubular tracing started at the beginning of the proximal tubules and ended at the macula densa of the same renal corpuscle. Tracing of the DVR of the juxta-medullary nephron started at the efferent arteriole and ended at the capillary network in the medulla.

## Results

In the present study, tubular digital tracing was performed for the purpose of investigating the establishment of the countercurrent loop-vessel arrangement during kidney morphogenesis, meanwhile for facilitating identification of the tubules and vessels appearing at stochastic sections of the developing and adult kidneys. By tracing tubule and vessels, their characteristics in running course, neighboring structures, location throughout the kidneys, as well as the constituent epithelial cell features were described in previous studies [8, 9, 20] and in the present study. By combination of tubular tracing with immunohistochemistry for AQP-1, AQP-2, and NKCC2, two pairs of loop-vessel countercurrent arrangement, proximal tubule-AVR and TAL-DVR in medulla were identified in the present study.

### Expression of AQP-2

**Fig 1:**
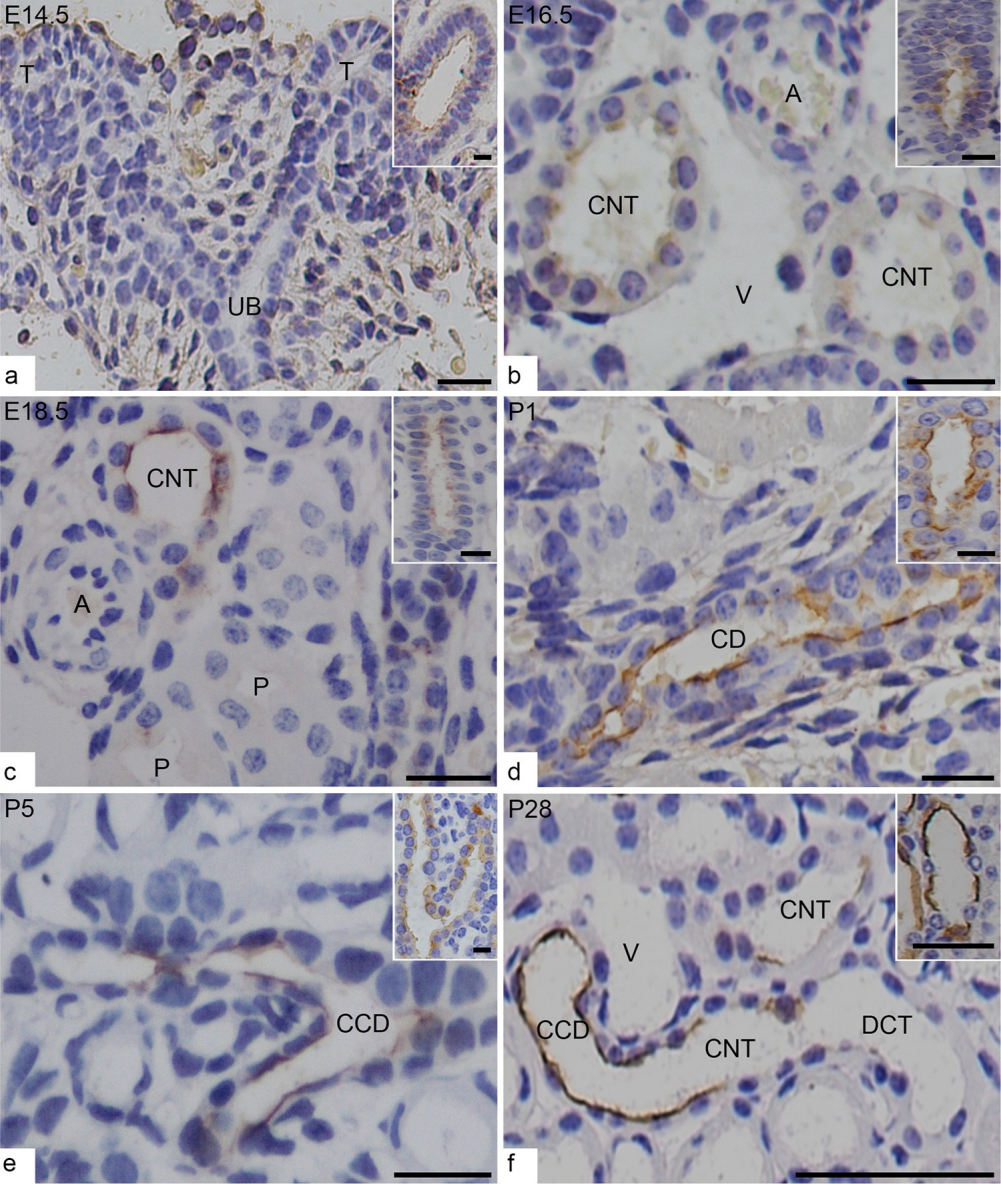
Expression of AQP-2 in the cortex and medulla of mouse developing and adult kidneys. (**a**) At E14.5, AQP-2 is not expressed in the upmost branched ureteric bud (UB) in the superficial cortex including the terminal tips (T) in nephrogenic zone, but uninterruptedly expressed in the main trunk of UB near the renal hilum (inset). (**b**-**c**) At E16.5 and E18.5, AQP-2 starts to discretely express in relatively mature connecting tubules (CNT) that have larger lumen and locates near the small artery (A) and vein (V), and UB trunks (insets). P for the mature proximal tubule. (**d**-**f**) After birth, AQP-2 discretely express in the branched cortical collecting duct (CCD) and CNT but not express in distal convoluted tubule (DCT). Inset are the UB or CD in deep medulla. Scales bars = 20 μm and 10 μm (insets).

As we already know that AQP-2 is expressed in principal cells distributed along connecting tubules (CNT), cortical and medullary collecting duct. In the present study, we noticed that the UB and their branches at the superficial cortex and their terminal tips at E14.5 did not express AQP-2 (Fig 1A), however, at the same time, AQP-2 was weakly and uninterruptedly expressed in the apical membrane and cytoplasm of the main UB trunk near the tip of the medulla (Fig 1A inset).

In the cortex, with the development, AQP-2 positive cells were often seen to constitute part of the connecting tubule epithelium before birth (Fig 1B and 1C) and the cortical collecting ducts after birth (Fig 1D–1F). The identification of the connecting tubule was based on the characteristics of a larger lumen at cross section with the discrete AQP-2 positive expression on apical part of the cells, and morphological features of the constituent cells such as apically located nuclei for distal tubule cells, as well as their location in the superficial cortex near small arteries and veins, which was consistent with the observations from tubular digital tracing.

### Expression of AQP-1 in cortex of developing mouse kidneys

**Fig 2:**
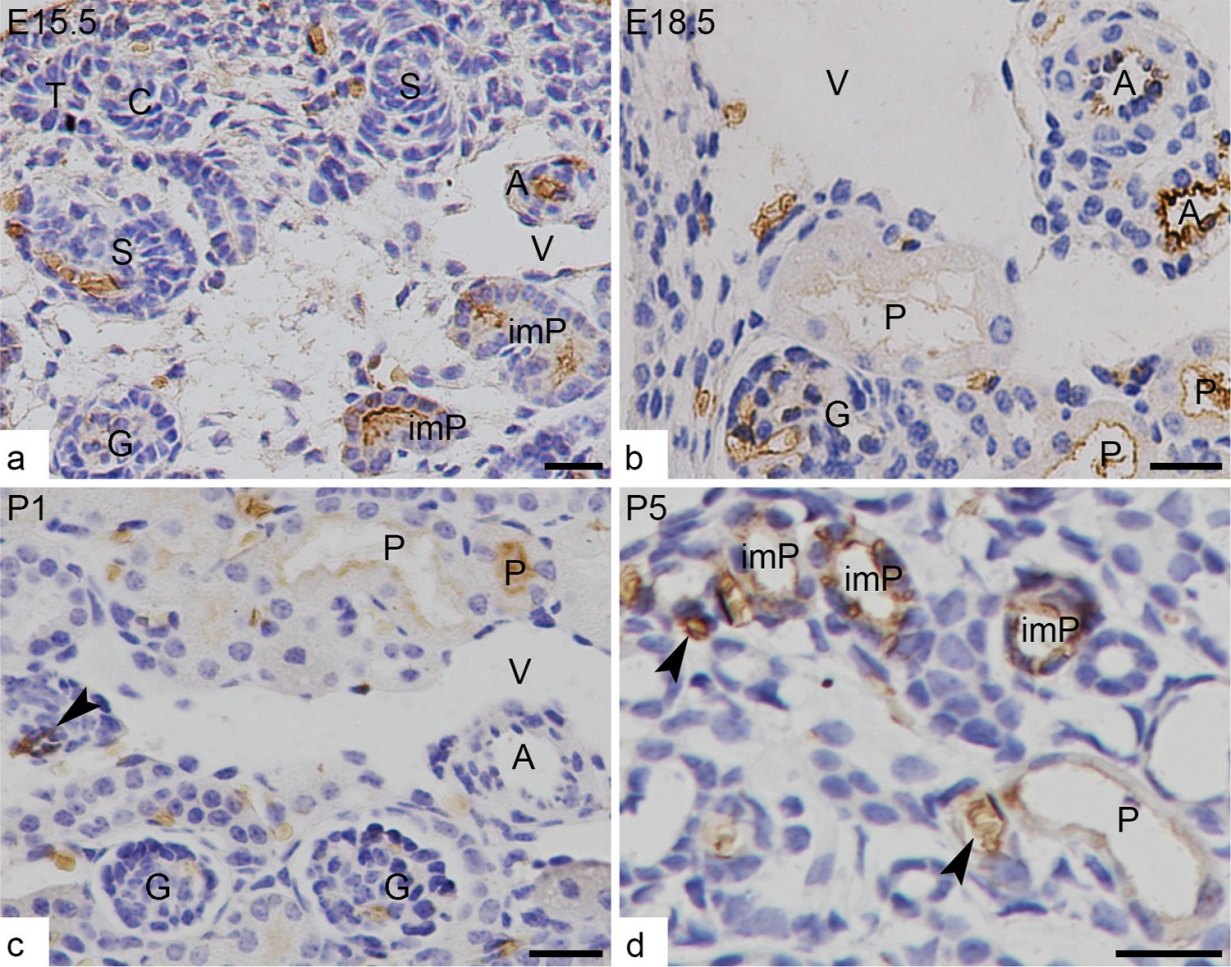
Expression of AQP-1 in the cortex of developing kidneys. (**a**) At E15.5, in the nephrogenic zone, the ureteric bud tip (T) and newly formed nephrons, Comma (C) -and S (S) -shaped bodies do not express AQP-1 except for those erythrocytes in the first cleft. Beneath the zone, AQP-1 is expressed at endothelium of small arteries (A) and at immature proximal tubules (imP) where the epithelial cells are arranged in a single layer and densely packed; (**b**) At E18.5, AQP-1 is still expressed at endothelium of small artery (A), and at mature proximal tubules (P) where the single layer of cells are pale stained and not densely packed; (**c and d**) At P1 and P5, AQP-1 continue to express at the immature (imP) and mature (P) proximal tubule, and at endothelium of arterioles (arrow heads) in cortex, but does not continue expressing at endothelium of artery (A); During development, veins (V) do not express AQP-1 at all. G for glomerulus. Scales bar = 20 μm.

Generally, AQP-1 was increasingly expressed in the structures, similar as those in the adult kidneys, i.e., apical and basolateral membrane of immature and mature proximal tubules, DTL, and DVR, from E14.5 to postnatal days (Fig 2). In addition, the extensive expression of AQP-1 in the vascular endothelium was observed in the artery system, including the main, arcuate, and interlobular arteries, as well as the arterioles that entered and left the glomerulus at the vascular pole. After birth, the expression was gradually confined to the arterioles (Fig 2A–2D). The expression of AQP-1 was lacking in the renal vein system.

In addition, AQP-1 was not detected immunochemically in the newly formed nephrons, such as comma- and S-shaped bodies.

### Expression of AQP-1 in the medulla of developing mouse kidneys

**Fig 3:**
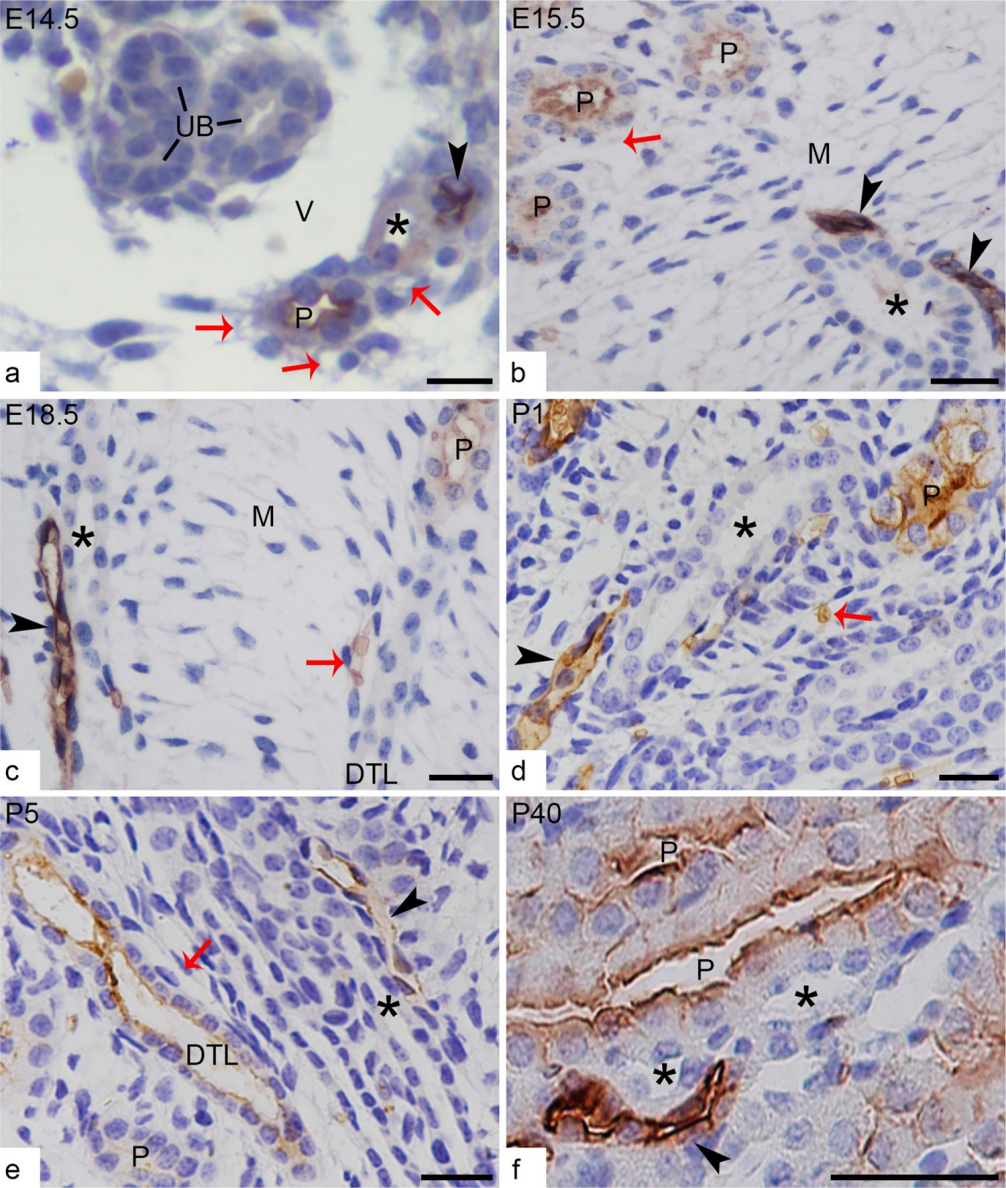
Expression of AQP-1 in developing and adult renal medulla, showing the countercurrent arrangement of TAL-DVR and DTL-AVR, as well as AQP-1 differentiated expression. (**a**) At the medullary level of E14.5 kidney, AQP-1 positive DVR (arrowhead) is located in close contact with AQP-1 negative TAL (asterisk). Thick walled proximal tubule (P) expressing AQP-1 at the brush border are surrounded by AQP-1 negative capillaries (red arrows). The UB trunk and branches do not express AQP-1 at all and stay separated from the tubule-vessel by a vein (V). (**b**-**c**) at E15.5 and E18.5, medullary mesenchymal cells (M) in interstitial space are arranged into a ladder-like framework, perpendicular to the pairs of the accompanied tubule-vessel. The AQP-1 positive proximal tubules (P) and descending thin limb (DTL) are surrounded by AQP-1 negative AVR or capillaries (red arrows). TAL (asterisks) are identified by its thick walled epithelium without AQP-1 expression and in proximity to DVR (arrowheads) expressing AQP-1. (**d**-**f**) After birth, more and more tubules and vessels extend into medulla and fill up the interstitial space. DVR (arrowheads) are still arranged parallel and close to TAL (asterisk), even in adult kidney (f), while AQP-1 positive proximal tubule (P) and its extension DTL are accompanied by elongated AVR or capillaries (arrowheads) containing AQP-1 positive erythrocytes. Scales bar = 20 μm.

Based on the differentiated expression of AQP-1 in the medulla, we discovered an intimate relationship of tubules and vessels (Fig 3A–3F). Early in E14.5 when the first generation of nephron loop of Henle grew into the juxta-medullary cortex and primary medulla, AQP-1 was expressed in the thick descending limb, i.e., the proximal straight tubules (PST), and descending thin limb (DTL). The two segments with AQP-1 expression were surrounded with either capillaries (round cross-sections) or in close contact with the AVR, which had a long and large lumen with a very thin and flattened epithelium. The capillaries and AVR did not express AQP-1 at all. At the same medullary level, not far from this pair of tubule and vessel, another pair of tubule and vessel with different AQP-1 expression revealed a similar intimate relationship and countercurrent arrangement, i.e., an AQP-1 positive DVR was found to locate in tight contact with the thick ascending limb (TAL). TAL were identified here by the microscopic features, i.e. cubic cells with nucleus apically located and striation border, and NKCC2 positive but AQP-1 negative expression. And the countercurrent arrangement of TAL-DVR and DTL-AVR were stable during mouse kidney development (Fig 3A–3F).

### Countercurrent arrangement of tubules and vessels in the medulla

**Fig 4:**
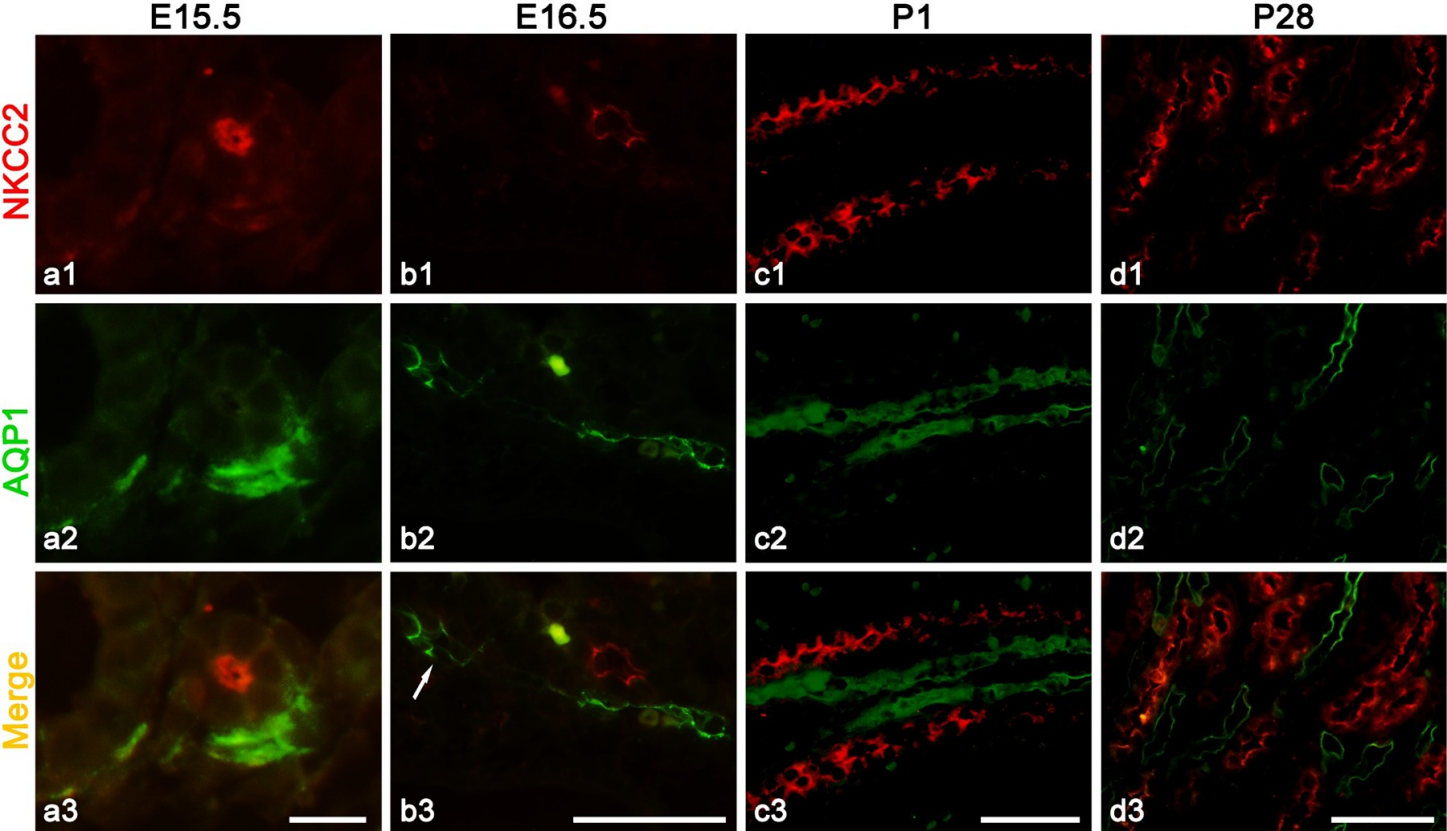
Double-labeled immunofluorescence for expression of NKCC2 in TAL and AQP1 in DVR or DTL, respectively, showing the countercurrent arrangement of TAL-DVR/DTL in developing and adult medulla. (a-d) NKCC2 (red) is expressed in the apical plasma membrane of thick walled TAL. AQP1 (green) is expressed in either DVR or DTL. From fetus to newborn (P1), TAL and DVR are always in close contact with each other in the medulla. In adult kidney medulla (P28), more tubules show up, and TAL run parallel to either DVR or DTL (arrow) both with AQP-1 positive expression. Scales bar = 10 μm (**a**) and 50 μm (**b**-**d**).

In order to verify that the tubule associated with DVR was TAL rather than UB or CD in the medulla, immunofluorescence studies was used to double-label NKCC2 for TAL and AQP-1 for DVR, which showed that AQP-1 positive blood vessels and NKCC2 positive tubules ran closely in the countercurrent form as soon as they extended into the medulla (Fig 4).

**Fig 5:**
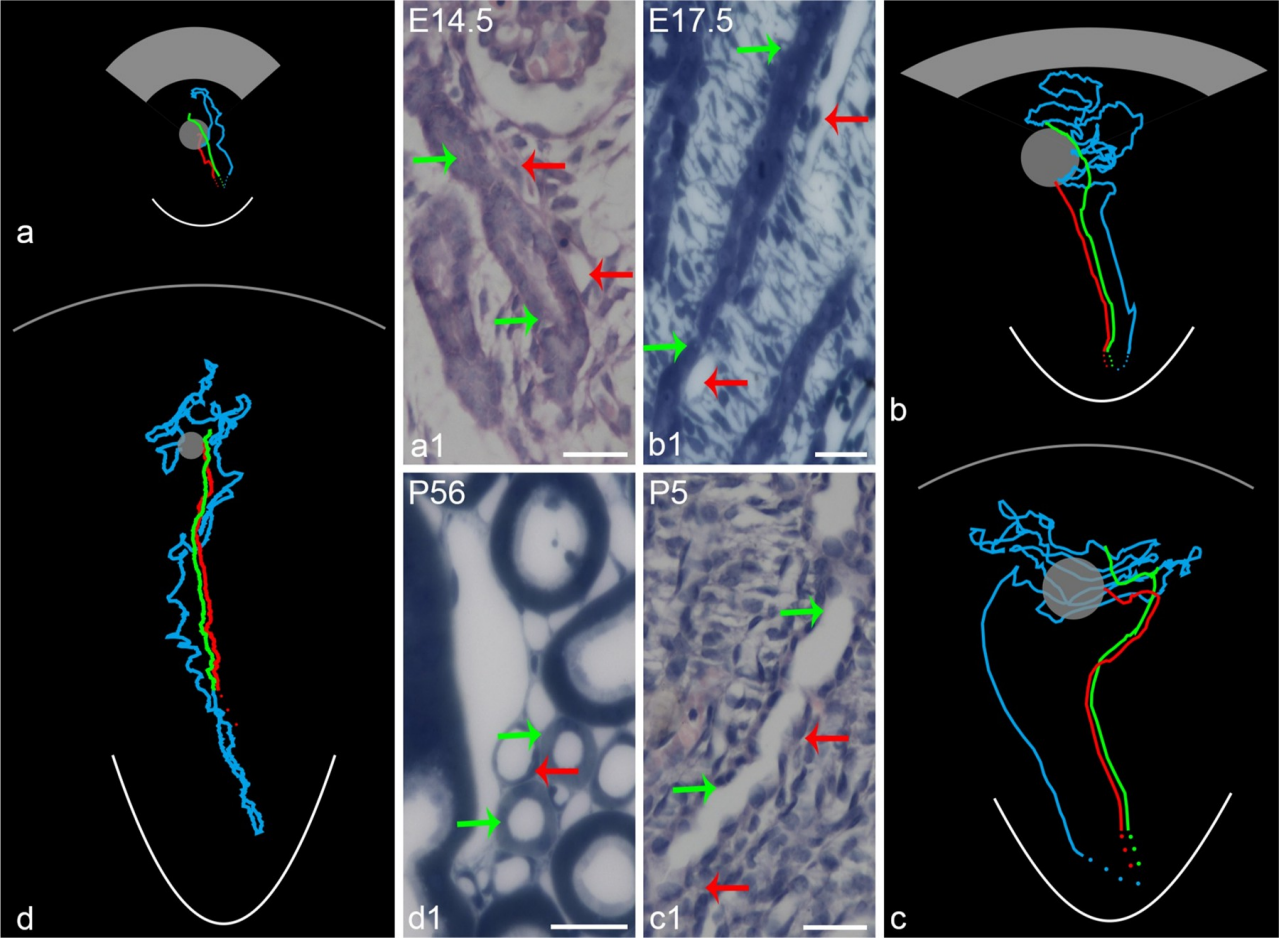
The countercurrent arrangement of the TAL and DVR is visualized in 3D representations and corresponding histological images. (**a**-**d).** The courses of TAL (green) and DVR (red) are 3D represented based on digital tracing of serial paraffin or epoxy sections, which correspond to the tubules and vessels pointed by green and red arrows respectively in **a1**-**d1.** Blue lines represent the course of the proximal tubules and thin limbs. The grey balls and the grey fan or arc for the glomeruli and nephrogenic zone (a and b) or renal capsule (c and d), respectively, and the white arc for the renal papilla. (a1-d1) The histological images were derived from serial paraffin sections with HE stain (a1 and c1) and serial epoxy sections with toluidine blue stain (b1 and d1). TAL and DVR in a1-d1 are identified by digital tracing, in which DVR tracing started at the efferent arterioles, TAL started from macular densa of same glomerulus as that of the efferent arterioles. Scales bar = 25 μm.

The computer-assisted three-dimensional visualization technology made it possible to trace the course of tubule-vessel and clarify the relationship between TAL and DVR. As result, during the embryonic period, the efferent arteriole extending into medulla as DVR was closely accompanied with their own TAL of early formed nephron. The countercurrent and intimate arrangement of DVR-AVR or capillaries and TAL-DVR persistently existed in developing kidneys and as a result in adult kidneys (Fig 5).

In summary, the tracing findings that DVR-TAL ran in a close contact in medulla further are consistent with that from immunohistochemistry on randomly selected paraffin sections.

## Discussion and conclusions

The 3D tubular tracing technique facilitates visualization of the course of the tubule and vessel in space in both previous adult mouse kidney studies and the present developing mouse kidney study [4, 16, 20]. Combining 3D visualization with tubular specific transporter immunohistochemistry for AQP-1, -2, and NKCC2, the present study demonstrated a gradually established tubule-vessel intimate relationship in the medulla at the early developing stage of the mouse kidney, as well as distribution of the transporters along the tubules and vessels.

First of all, AQP-2 was differentially expressed in the collecting duct system, i.e. from uninterrupted expression in UB trunk at the early stage to discrete expression with time, and from negative expression in cortical branches and the tips of UB to positive and interrupted expression in connecting tubules and collecting ducts with kidney maturation. The finding supports the hypothesis that AQP-2-positive cells in the UB trunk are capable of differentiating to intercalated cells [21–23]. While, the negative expression of cortical branches and the tip of UB may represent an inductive role, rather than the function of water transportation at an early stage of nephrogenesis.

Secondly, an extensive expression of AQP-1 in the vascular endothelium was observed to exist in the renal main arteries, arcuate, and interlobular arteries before birth and vanish after birth. One exception to it was that the expression for AQP-1 in arterioles existed through whole embryonic days to adulthood, including the efferent arterioles of juxtamedullary nephrons and its continuation, DVR in the medulla. A similar observation in rats has been reported and is supposed to be related to fluid equilibrium or the regulation of growth and branching of the arterial vascular tree during kidney development [24].

Finally, the most important finding of this study is that, early at E14.5, two kinds of pairs of tubule-vessel were observed to be closely associated by immunohistochemistry for AQP-1, which was further confirmed by digital tracing and found that the tubule-vessel arranged in countercurrent pattern grew and extended from the cortex into the medulla with time. As nephrogenesis constantly occurred, more and more pairs of such countercurrent tubule-vessel extended and ran in different regions of the medulla. Functionally, one kind of pair consisted of AQP-1 positive DVR and AQP-1 negative but NKCC2 positive TAL, the other pair was AQP-1 positive proximal straight tubule and DTL and AQP-1 negative AVR. Therefore, the specific spatial arrangement might imply less physical accompany of the tubule and vessel during tubulogenesis than establishing a structural basis for the efficient exchange for water and other electrolytes between the tubule and vessel. The pair of the TAL-DVR observed at the early stage of kidney development are supposed to be the longest looped nephrons and therefore will eventually grow into the outer medulla in the adult kidney, running mainly in the outer part of the vascular bundle in the inner stripe of the outer medulla in mice [4]. While the TAL of SLN are from late formed nephron will then stop at various levels of the inner stripe of the outer medulla, running in the interbundle region, surrounded by fenestrated capillaries. Therefore, the paired TAL-DVR countercurrent arrangement may not represent the population of the TAL of short-looped nephrons.

In summary, in addition to a detailed description of the distribution of AQP-1 and AQP-2 in developing kidneys, the present study exhibits the countercurrent arrangement of tubule-vessels in early-developed medulla, which reflects an early establishment of the countercurrent exchange parallel to morphogenesis of medulla, as well as water and electrolyte transportation. Based on the findings, the molecular mechanisms driving the formation of the tubule-vessel countercurrent arrangement need to be further studied with e.g. gene manipulations, so does the physiological function of the countercurrent arrangement of the tubule-vessel with water and solute transporters in especially formation of a concentrated urine, which are unable to be fulfilled with present study method.
